# Structure-based identification of novel FAK1 inhibitors using pharmacophore modeling, molecular dynamics, and MM/PBSA calculations

**DOI:** 10.1038/s41598-025-23203-8

**Published:** 2025-11-11

**Authors:** Amirhossein Hajipasha, Nilofar Ghaffari Cherati, Mohammad Darzi, Seyedeh Sana Nateghi, Seyed Arshia Sadat Mohsenian, Mahla Noorzaei, Fatemeh Kashiri Dinaki, Mohammad Halimi

**Affiliations:** Department of Biology, Babol Branch, Islamic Azad University, Babol, Iran

**Keywords:** FAK1 inhibitor, Pharmacophore modeling, Docking, MD simulation, MM/PBSA, Cancer, Computational biology and bioinformatics, Drug discovery

## Abstract

Focal adhesion kinase 1 (FAK1) is a non-receptor tyrosine kinase involved in cancer metastasis and tumor progression. Due to its role in regulating cell migration and survival, FAK1 is considered a promising target for cancer therapy. While large chemical databases such as ZINC offer a wide variety of molecules, many of them have not yet been explored as potential FAK1 inhibitors.In this study, we applied several computational methods to identify new inhibitors of the FAK1 kinase domain. Pharmacophore models were built based on the FAK1–P4N complex (PDB ID: 6YOJ), and the most statistically reliable model was used to screen compounds from the ZINC database. Hits were first docked using AutoDock Vina in PyRx, and seventeen compounds with acceptable pharmacokinetic properties and low predicted toxicity were selected for more precise docking via SwissDock. Four promising candidates—ZINC23845603, ZINC44851809, ZINC266691666, and ZINC20267780—were chosen for molecular dynamics (MD) simulations using GROMACS. The stability and behavior of each protein–ligand complex were examined, and binding free energies were calculated using the MM/PBSA method. Among them, ZINC23845603 showed strong binding and interaction features similar to the known ligand P4N. Given its favorable binding energy and pharmacokinetic profile, ZINC23845603 may be a good candidate for further experimental studies targeting FAK1.

**Supplementary Information:**

The online version contains supplementary material available at 10.1038/s41598-025-23203-8.

 One of the most important proteins in cancer metastasis is Focal adhesion kinase 1 (FAK1). This protein is a non-receptor tyrosine kinase and has been associated with metastasis in breast cancer, ovarian cancer, prostate cancer, head and neck squamous cell carcinoma, melanoma, and several other cancer types. Integrins and other signaling molecules interact with this protein at focal adhesion sites. This interaction enables signal transmission from the matrix to the cell interior^1,2^. By performing these functions, this protein has become a key component in the transduction of signals that promote tumorigenic behaviors, including enhanced cell motility and invasion. Accordingly, FAK1 is a promising target for drug design^3,4^. Human FAK1 is a large protein with three domains and 1,052 residues (Fig. 1). The N-terminal domain is named FERM, which regulates interactions and activities. The C-terminal domain, or FAT domain, enables attachment to focal adhesions, and the central domain, or the kinase domain, phosphorylates target proteins^5^.

Most FAK1 inhibitors have been designed for the kinase domain. The best known of these inhibitors include Defactinib, which has been evaluated in clinical trials for solid tumors and GSK2256098 which has undergone clinical trials, particularly in mesothelioma^6^. P4N is another inhibitor of the kinase domain, which is in the preclinical phase^7^. P4N binds to the ATP-binding pocket of FAK1 and blocks its autophosphorylation and downstream signaling, thereby reducing cancer cell proliferation and tumor progression^8–11^. In recent years, more inhibitors like BI 853,520 have been studied, which is a highly selective FAK inhibitor that has shown good results in mouse models for reducing tumor growth in adenocarcinoma, but it still has issues with long-term effects in humans due to poor bioavailability and clearance rates^12^. Another example is BSJ-04–146, a PROTAC-based inhibitor that not only blocks the kinase activity but also degrades the FAK protein itself, leading to better control over cancer cell migration in lab tests compared to traditional inhibitors like Defactinib^8^. However, many of these inhibitors face problems such as limited selectivity, where they affect other kinases like PYK2 or Src, or poor pharmacokinetic properties that make them hard to absorb or stay in the body long enough^13^. For example, Defactinib has been tested in phase II trials for ovarian cancer but showed only moderate improvements in patient survival rates, partly because of resistance development over time or its inability to block FAK’s scaffolding roles, which are important for cancer progression without involving phosphorylation^14^. These scaffolding roles help cancer cells adhere to the extracellular matrix, and inhibitors targeting only the kinase activity often fail to disrupt them fully^15^.

Previous researches on FAK1 inhibition have utilized both computational and experimental methods to identify and test new compounds. On the computational side, approaches such as pharmacophore modeling have been applied to identify novel inhibitors by mapping key interactions in the FAK1 binding pocket. For example, some studies used the FAK1-P4N complex from PDB to screen for allosteric inhibitors that bind outside the ATP site, which could avoid selectivity issues seen with ATP-competitive inhibitors^16^. Molecular docking has been widely used to predict how small molecules fit into the kinase domain, for example, in virtual screening of databases like PubChem to find hits with low binding energies, and then molecular dynamics (MD) simulations have been employed to check the stability of these complexes over time, revealing how flexible loops in FAK change during binding^17^. These computational methods help to reduce the cost and time compared to traditional drug discovery. For instance, a recent study combined machine learning with docking to identify FAK inhibitors for breast cancer, finding compounds with better binding affinity than earlier hits^18^. Another computational work used free energy calculations to study the dynamic behavior of FAK1 with inhibitors like TAE226, showing how specific residues like Leu553 stabilize the complex^16^. Experimentally, in vitro kinase assays have been common to measure how well inhibitors block FAK phosphorylation, often using cell lines from cancers like prostate or lung to test cell death or migration reduction. For example, studies with GSK2256098 in mesothelioma cell lines demonstrated reduced cell invasion but highlighted the need for combination therapies with drugs like trametinib to overcome resistance^19^. In vivo models, such as injecting tumor cells into mice and treating with inhibitors, have shown how these compounds can slow metastasis in real biological systems, like in pancreatic cancer models where FAK inhibitors reduced tumor spread to the liver^20^. Despite these efforts, there is still a need for new inhibitors because many existing ones have limitations in selectivity or do not cover all functions of FAK, like its non-kinase roles. Also, computational studies often focus on small chemical libraries without full ADME and toxicity checks, while experimental approaches can be expensive and time-consuming, leaving gaps in finding safe and effective candidates from large databases like ZINC^21,22^.

In-silico approaches like virtual screening have become a part of drug discovery process which enables the researchers to identify potential drug candidates among large chemical libraries and reduce the time and expense of drug discovery^22,24,24^. Investigating the interaction between small molecules and target proteins and evaluating their binding affinity has become possible using molecular docking approaches. By using molecular dynamics (MD) simulations and free energy calculations, the conformational changes of the target protein and small molecule over time can be monitored, and the stability of the complex can be predicted with high accuracy, as well as key residues essential for ligand binding can be pinpointed^25,27,27^. In this study, we used the P4N-FAK1 complex to develop a structure-based pharmacophore model to highlight the most important interactions. The pharmacophore model was subsequently validated and employed to conduct a virtual screening of the ZINC database in search of potential FAK1 inhibitors. After performing docking and evaluating binding affinities, the ADME properties and toxicity profiles of the most promising compounds were analyzed using online tools. Ultimately, MD simulations and free energy analysis were performed to evaluate the stability and interaction of the chosen FAK1-inhibitor complexes, facilitating the identification of potential drug candidates that are both effective, safe, and stable.

## Materials and methods

### Preparation of the structure

The co-crystal structure of the FAK1 kinase domain in complex with P4N (6-[4-(3-methanesulfonyl-benzylamino)−5-trifluoromethyl-pyrimidin-2-ylamino]−3,4-dihydro-1 H-quinolin-2-one) (PDB ID: 6YOJ), was obtained from the Protein Data Bank (WWW.rcsb.org)^28^. This structure contains residues 411–689 of FAK1. The structure has a resolution of 1.36 Å, a free R-value of 0.198, and a work R-value of 0.171. However, residues at positions 570–583 and 687–689 were missing in the structure. Therefore, the missing residues were modeled using the MODELLER 9.25 software via the Chimera interface^29,31,31^. Five models were generated, and the model with the lowest zDOPE score was chosen as the reference structure for subsequent analysis.

### Pharmacophore modeling

In structure-based pharmacophore modeling, the intermolecular interactions between the receptor and the ligand are studied, and the interactions that play the most important role are identified. Pharmit (http://pharmit.csb.pitt.edu) is a web-based tool that creates pharmacophore models from receptor-ligand complexes. On this website, the user can also create personalized chemical libraries of decoy and active compounds for the purpose of validation. Finally, large chemical libraries can be screened by the validated pharmacophore. In this study, the FAK1-P4N complex was uploaded to Pharmit, and the critical pharmacophoric features involved in the formation of this complex were identified. Initially, Pharmit detected eight pharmacophoric features. Subsequently, the user generated 6 pharmacophore models, each containing five or six features. The models were used to evaluate both active and decoy libraries, and the one with the highest validation performance was chosen for additional screening of chemical libraries.

### Pharmacophore validation

A pharmacophore model must be validated before it can be used in the process of virtual screening. To this end, two groups of compounds are needed, including actives, s i.e., compounds that bind to the target with sufficient affinity and activity, and decoys, i.e.compounds that fail to bind to target. A proper pharmacophore model should be able to detect the most number of actives (high sensitivity) and the least number of decoys (high specificity)^32,33^. In this study, 114 active and 571 decoys for FAK1 were downloaded from the DUD-E database (http://dude.docking.org/) as of December 26, 2024. Directory of Useful Decoys - Enhanced (DUD-E) is a website that provides active and decoys for a total number of 102 biological targets^34^.

For the evaluation of pharmacophore models, the decoy and active inhibitors of FAK1 were uploaded into Pharmit and then screened by 6 different pharmacophore models. After screening of these libraries, several statistical metrics were calculated. These included sensitivity and specificity, as defined by Eqs. (1) and (2). Yield of active compounds (YA), also referred to as recall, the enrichment factor (EF), and the goodness of hit (GH) were determined based on Eqs. (3), (4), and (5), respectively. These indicators were determined using the corresponding equations presented below.

The sensitivity, also known as the true positive rate, is calculated as:

1 $$Sensitivity = (Ha / A)\times100$$

The specificity, or true negative rate, is given by:

2 $$Specificity = (True negatives / Decoys)\times100$$

The yield of actives (YA), or recall, is calculated as:

3 $$YA (Recall) = (Ha / Ht)\times100$$

The enrichment factor (EF) is computed as:

4 $$EF = YA / (A / D$$

Finally, the goodness of hit (GH) is determined using the formula^35,36^.

5 $$GH = (Ha(3A + Ht) / 4HtA) \times (1 - (Ht - Ha) / (D - A))$$

### Virtual screening

The best pharmacophore (Pharm_1) was used for screening the ZINC databese, including 13,127,550 molecules. The following filters were also used in screening: molecular weight (MW) limited to 500 Da or lower, hydrogen bond donors (HBDs) capped at 5, hydrogen bond acceptors (HBAs) restricted to a maximum of 10, an octanol/water partition coefficient (A log P) not surpassing 5, rotatable bonds limited to 10 or fewer, and a polar surface area of no more than 140 Å².

The result of screening was saved in Structure Data File (SDF) format and used for molecular docking using AutoDock Vina implemented in PyRx 0.8 (https://pyrx.sourceforge.io/downloads)^30^. The following steps were done before docking: Gasteiger partial atomic charges and polar hydrogen atoms were assigned to each molecule, followed by energy optimization to refine their geometries. Structural conversion to the PDBQT file format was accomplished using Open Babel’s functionality embedded within PyRx. A docking grid was defined to align with the FAK1-P4N interaction site, with a cubic volume of 23 × 23 × 23 Å³ centered at coordinates X = −16.605, Y = −2.074, Z = 20.311.

### ADME and toxicity prediction

Effective impact on the specified therapeutic target is a fundamental requirement; however, it is not the only criterion. A promising drug candidate must also exhibit favorable ADME properties^37^. Estimating the ADME characteristics of compounds is thus critical for the identification and optimization of hits. To achieve this goal, the ADME characteristics of the selected hit compounds were evaluated using SwissADME, an online platform designed to compute various ADME-related parameters, physicochemical attributes, and additional descriptors relevant to drug-like molecules^37,38^. A crucial aspect of the drug discovery process is evaluating the potential toxicity of compounds. In this study, ProTox-III was employed for this purpose^39^. ProTox-III is a virtual laboratory that allows for the prediction of various toxicity models.

### Molecular Docking study

Docking studies were performed using SwissDock^40,41^. SwissDock relies on the EADock DSS docking engine, which performs local docking through a specific algorithm. Initially, numerous potential binding conformations are created within a user-defined region. These conformations are then assessed using grid-based CHARMM energy estimations, and those with the most favorable energies are selected and grouped into clusters^40,41^. Before docking, ligand and water molecules were removed and hydrogen atoms were added to enhance docking accuracy. The protein was rigid and different ligand conformations were docked into the binding site. Prior to docking, ligands were subjected to energy minimization using Avogadro version 1.2.0, in order to resolve any atomic clashes and generate an appropriate initial conformation^42^. The minimization process employed the Universal Force Field (UFF) in combination with the steepest descent algorithm. Visualization and analysis of ligand–receptor interactions were performed using Discovery Studio Visualizer 2016 (Accelrys Inc., San Diego, CA, USA) and UCSF Chimera version 1.14^43^. Different ligand poses were ranked according to their binding energy. Lower energy values indicate more stable interactions.

### Molecular dynamics simulation study

MD simulations were performed using the GROMACS 5.1.2 software suite installed on the Ubuntu 18.04.5 LTS platform^44^. The topology file of the protein was created using the pdb2gmx utility, employing the CHARMM36 force field. The topology files and force field parameters of the ligands were generated using SwissParam^45^. The ligand and protein were first converted from.pdb to.gro format and then combined as a complex manually by using Notepad^++^. In the next step, the topology file of the protein was modified as follow: the.itp file of the ligand that was made by SwissParam was added, andthe ligand name was also added at the end of the topology file. The protein-ligand complex was then placed in a cubic box, ensuring a minimum distance of 1.0 nm between the complex and the box boundaries. In the next step, a total number of 11,525 water molecules as the SPC216 water model were added into the box. Then, the system was electrically neutralized by substituting seven of these water molecules with Na^+^ ions. The steepest descent algorithm, with a maximum of 50,000 steps, was executed for energy minimization of the system. Structural relaxation was ensured until the highest force acting within the system fell below 10.0 kcal/mol. For NVT equilibration, a v-rescale thermostat was employed to regulate the temperature at a steady 300 K. This phase lasted 500 ps with a coupling constant of 0.1 ps, allowing the system to reach thermal equilibrium. For NPT equilibration, the Berendsen pressure coupling algorithm was applied with a coupling constant of 5.0 ps over a period of 1,000 ps. The particle-mesh Ewald (PME) method was utilized to account for long-range electrostatic interactions. A cutoff distance of 1.0 nm for electrostatics and 1.2 nm for van der Waals interactions was incorporated. Following system stabilization, each compound was subjected to three independent 100-nanosecond molecular dynamics simulations. Frames were extracted from the trajectory at intervals of 10 ps, yielding a total of 10,000 frames. After successfully completing the molecular dynamics simulation, the protein-ligand complex was repositioned at the center of the simulation box to ensure proper alignment for post-simulation analysis. A comprehensive set of analyses was then performed to assess the stability and dynamic behavior of the system. These analyses included calculating the root mean square deviation (RMSD) to monitor structural changes over time, determining the radius of gyration (gy) to evaluate overall compactness, and quantifying the number of hydrogen bonds both within the protein and between the ligand and protein throughout the simulation. Additionally, the root mean square fluctuations (RMSF) of backbone residues were analyzed to identify flexible and rigid regions within the protein structure.

### Free binding energy calculations

The binding free energy of the protein-ligand complex was determined using the molecular mechanics Poisson-Boltzmann surface area (MM/PBSA) approach, without entropy contributions. The binding free energy (ΔGbind​) is expressed as the sum of the molecular mechanics interaction energy (MMIE) and solvation energy (SE):

$$\triangle {G_{{\text{bind}}}}{\text{ = MMIE + SE}}$$

Where MMIE represents the direct interaction energy between the protein and ligand, comprising:

$${\text{MMIE = }}{{\text{E}}_{{\text{vdW}}}}{\text{ + }}{{\text{E}}_{{\text{elec}}}}$$

MMIE = E_vdW_ + E_elec_.

where E_vdW_​ is the van der Waals interaction energy, and E_elec_​ is the electrostatic interaction energy.

SE accounts for the solvation effects and is divided into polar and nonpolar contributions:

(1) SE = PSE + SASA energy.

Polar solvation energy (PSE) is derived from the Poisson-Boltzmann equation:

(2) PSE = PSE_complex_ − (PSE_protein_ + PSE_ligand_).

Nonpolar solvation energy (SASA energy) is estimated based on solvent-accessible surface area (SASA):

(3) SASA_energy_ = SASA_complex_ − (SASA_protein_ + SASA_ligand_).

Binding free energy calculations were performed using 200 snapshots extracted at 100 ps intervals from the final 20 ns of the molecular dynamics trajectory. These snapshots provided a representative sampling of the protein-ligand interactions in the stabilized phase of the simulation.

## Results and discussion

### FAK1-P4N pharmacophore feature mapping

According to Fig. 2, there are 4 hydrogen bonds between P4N and FAK1 kinase domain residues, including Cys95 and Asp157. Carbon-hydrogen bonds with much fewer energy were also detected with Glu93 and Gly156. Residues Met92, Leu146, Ala45, and Ile21 are involved in hydrophobic interactions, including π-alkyl and alkyl interactions. π-sigma interactions are formed between P4N and with Leu94 and Leu146. For constructing the pharmacophore, this complex was used in Phramit. Eight pharmacophoric features were identified in this step. Subsequently, 6 models, each comprising five or six features, were constructed. Active and decoy libraries were screened by all models, and one model with the best scores (Pharm_1) was chosen for screening the ZINC library. The results of the pharmacophore validation process are presented in Fig. 3. The three-dimensional configuration and spatial coordinates (x, y, z) of Pharm_1 are shown in Fig. 4. Importantly, Pharm_1 integrated four out of the six hydrogen bonds and two of the four hydrophobic interactions identified in the FAK1-P4N complex. These non-covalent interactions are detailed as follows: three hydrogen bonds involving N16–Cys95, N23–Cys95, and O9–Asp157, along with two hydrophobic interactions between aromatic rings and the residues Ile21 and Leu146. By these interactions, P4N is stabilized in the binding pocket of FAK1.

**Fig. 1:**
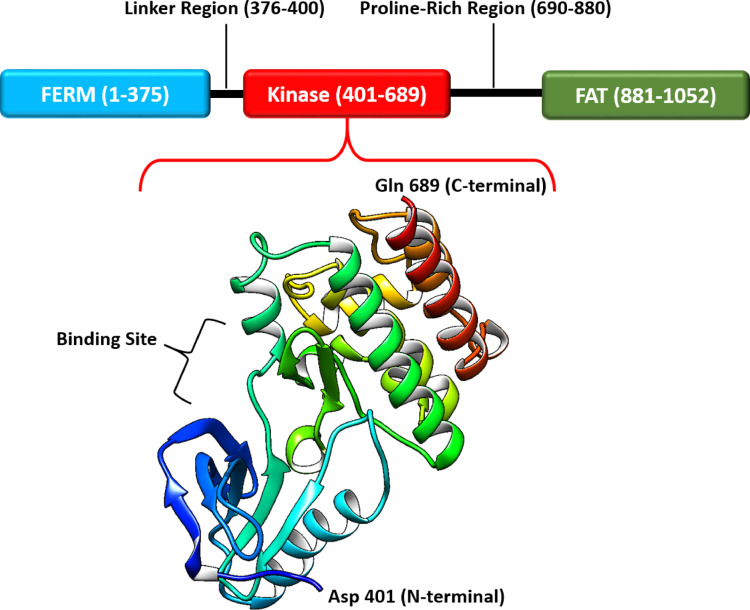
Human FAK1, comprising 1,052 amino that includes three main domains: the FERM domain at the N-terminus; the kinase domain in the central region; and the FAT domain at the C-terminus, which enables attachment to focal adhesions. Structure visualized using Chimera 1.14 based on PDB ID: 6YOJ.

**Fig. 2:**
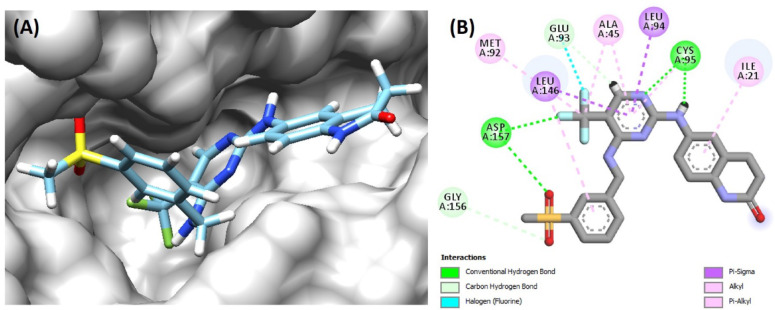
(**A**) Orientation of P4N in complex with FAK1. (**B**) Non-bonding interactions of P4N within the binding site of FAK1.

**Fig. 3:**
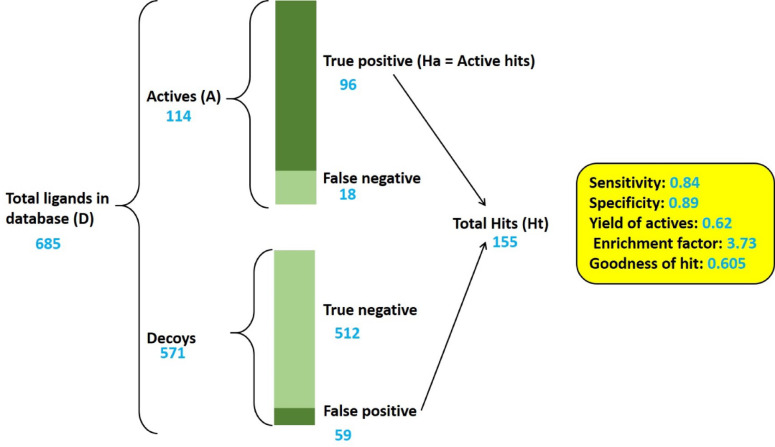
Overview of the pharmacophore validation process and the results.

**Fig. 4:**
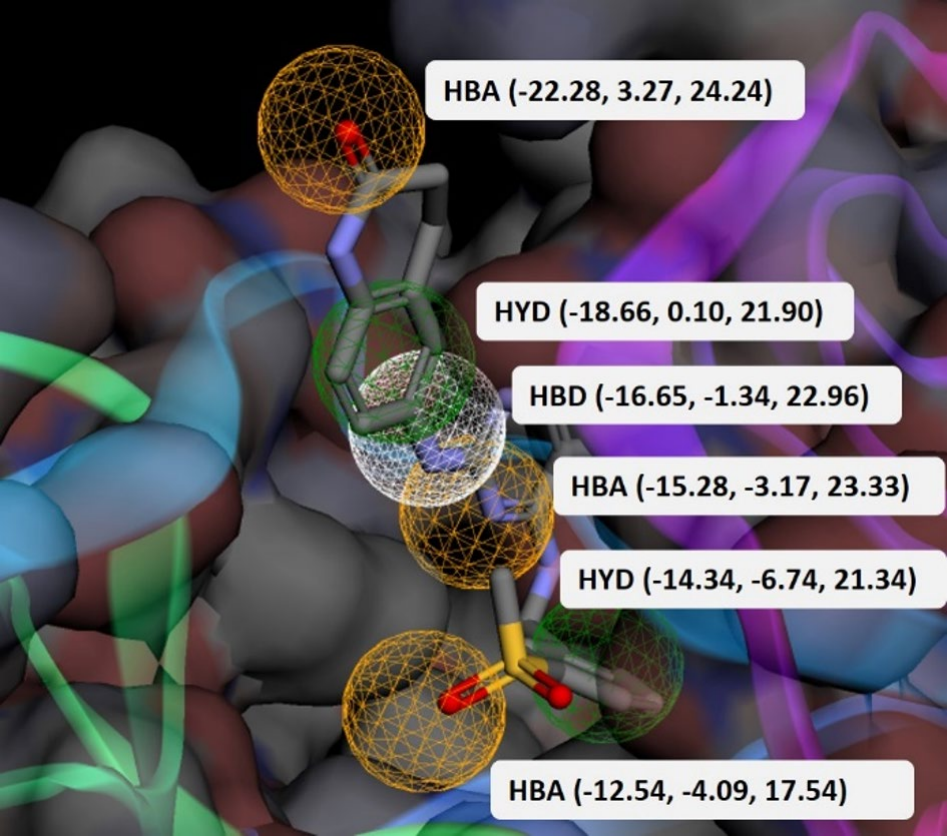
Structure of Pharm_1, with functional groups labeled: HBA (Hydrogen Bond Acceptor), HBD (Hydrogen Bond Donor), and HYD (Hydrophobic).

### Virtual screening

Screening of the ZINC library was performed in two steps. In the first step, the Pharm_1 pharmacophore model was used for screening. In this step, filtration criteria like Lipinski’s rule of five and Veber’s parameters for drug-likeness were incorporated as well. This screening yielded 3,102 structurally compliant compounds. In the second step, the binding energetics of these compounds at the FAK1 catalytic domain were calculated by AutoDock Vina and using PyRx. Finally, 95 top-ranked candidates with the strongest calculated binding affinities (lowest free energy values) were selected for ADME and toxicity prediction.

### ADME and toxicity prediction

For a compound to be drug-like, binding to the biological target is essential, but having suitable pharmacokinetic properties and low toxicity is also important too. In this study, two reliable tools, including SwissAdme and ProTox-III, were employed for the evaluation of these properties. From 95 investigated compounds, 17 compounds had favorable pharmacokinetic properties and low toxicity. Tables 1, 2 and 3 show the pharmacokinetic and toxicity data of the top four compounds that were chosen after extensive docking.

**Table 1:** Molecular properties of the selected compounds.

Compound	Formula	iLogP	TPSA	Heavy Atoms	MW	nHBA^a^	nHBD^b^	nRotb
ZINC23845603	C23H25N3O7	1.67	123.27	33	455.46	7	2	10
ZINC44851809	C22H28N2O6S	2.51	102.55	31	448.53	6	1	9
ZINC266691666	C21H24N4O4	1.36	122.55	29	396.44	4	4	9
ZINC20267780	C19H23FN4O4	1.24	90.98	28	390.41	6	2	7

^a^ Number of hydrogen bond acceptors.

^b^ Number of hydrogen bond donors.

**Table 2:** Predicted ADME properties of the selected compounds using SwissADME.

Compound	GIA^a^	BBBP^b^	CYP1A2 inhibitor	CYP2C19 inhibitor	CYP2C9 inhibitor	CYP2D6 inhibitor	CYP3A4 inhibitor	Lipinski	Log S
ZINC23845603	High	No	No	No	Yes	No	Yes	Yes	−3.86
ZINC44851809	High	No	No	Yes	Yes	Yes	Yes	Yes	−4.09
ZINC266691666	High	No	No	No	No	No	No	Yes	−2.53
ZINC20267780	High	No	No	No	No	No	No	Yes	−2.87

^a^ Gastrointestinal absorption.

^b^ Blood–brain barrier permanent.

**Table 3:** Predicted toxicity risks of the selected compounds using ProTox-II.

Compound	Hepatotoxicity	Carcinogenicity	Mutagenicity	Cytotoxicity	Predicted LD50	*P*.T.C ^a^
ZINC23845603	Inactive (0.68)	Inactive (0.62)	Inactive (0.74)	Inactive (0.67)	1000 mg/kg	4
ZINC44851809	Inactive (0.64)	Inactive (0.57)	Inactive (0.60)	Inactive (0.66)	1500 mg.kg	4
ZINC266691666	Inactive (0.81)	Inactive (0.61)	Inactive (0.63)	Inactive (0.65)	800 mg/kg	4
ZINC20267780	Inactive (0.77)	Inactive (0.52)	Inactive (0.63)	Inactive (0.77)	1000 mg/kg	4

^a^ “Predicted Toxicity Class” is a number from 1 to 6 that higher numbers indicate lower toxicity..

### Molecular Docking study

**Fig. 5:**
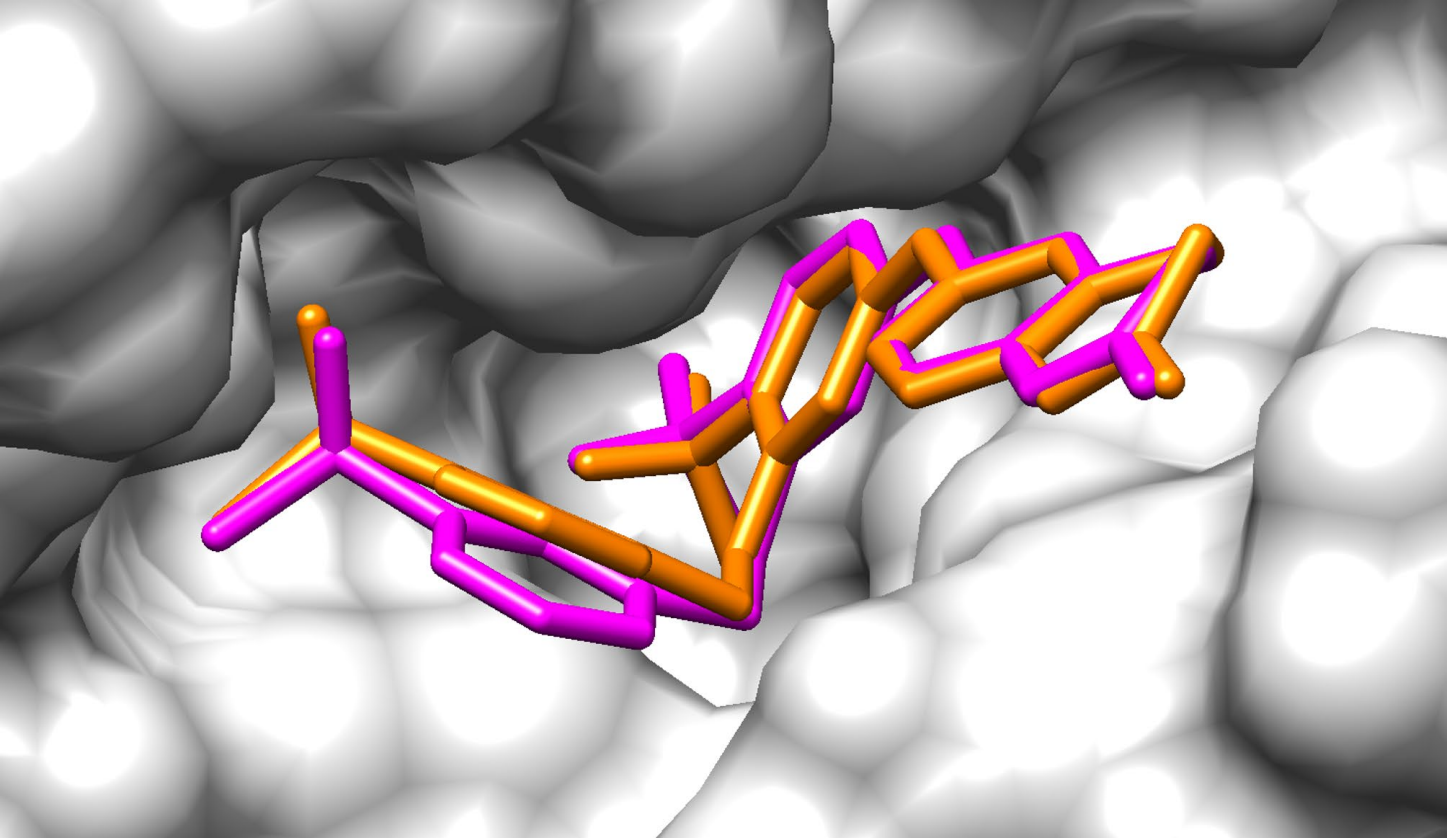
Superimposition of P4N from X-ray crystallography (orange) and the docking simulation (pink) within the binding site of FAK1.

To obtain the best pose of the ligands in the binding site of FAK1 kinase domain, high-precision docking was performed by SwissDock. To validate the docking protocol the reference ligand i.e., P4N was redocked in its binding site and root-mean-square deviation (RMSD) between the top-ranked conformation of P4N (affinity: −10.363 kcal/mol), and the co-crystallized pose was calculated. The RMSD of 1.33 Å confirmed strong spatial alignment between predicted and experimental ligand orientations (Fig. 5).

**Fig. 6:**
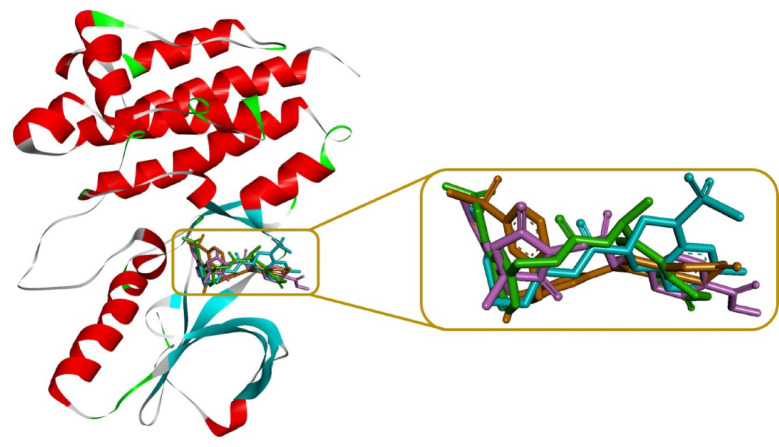
Compounds with the highest binding energies, alongside P4N, within the FAK1 binding site.

**Fig. 7:**
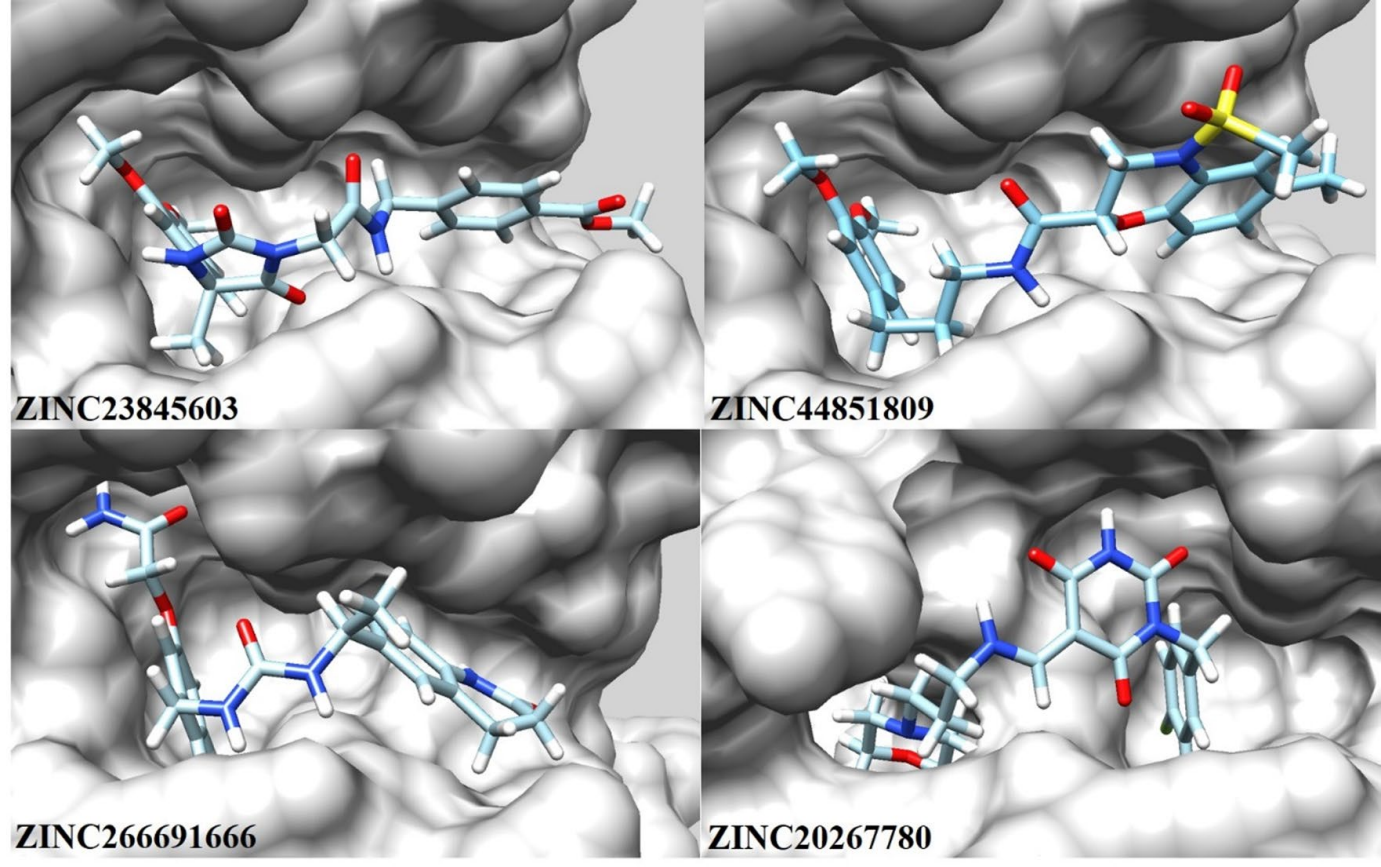
Optimal binding poses of the selected compounds within the FAK1 binding site, as determined by docking studies.

**Fig. 8:**
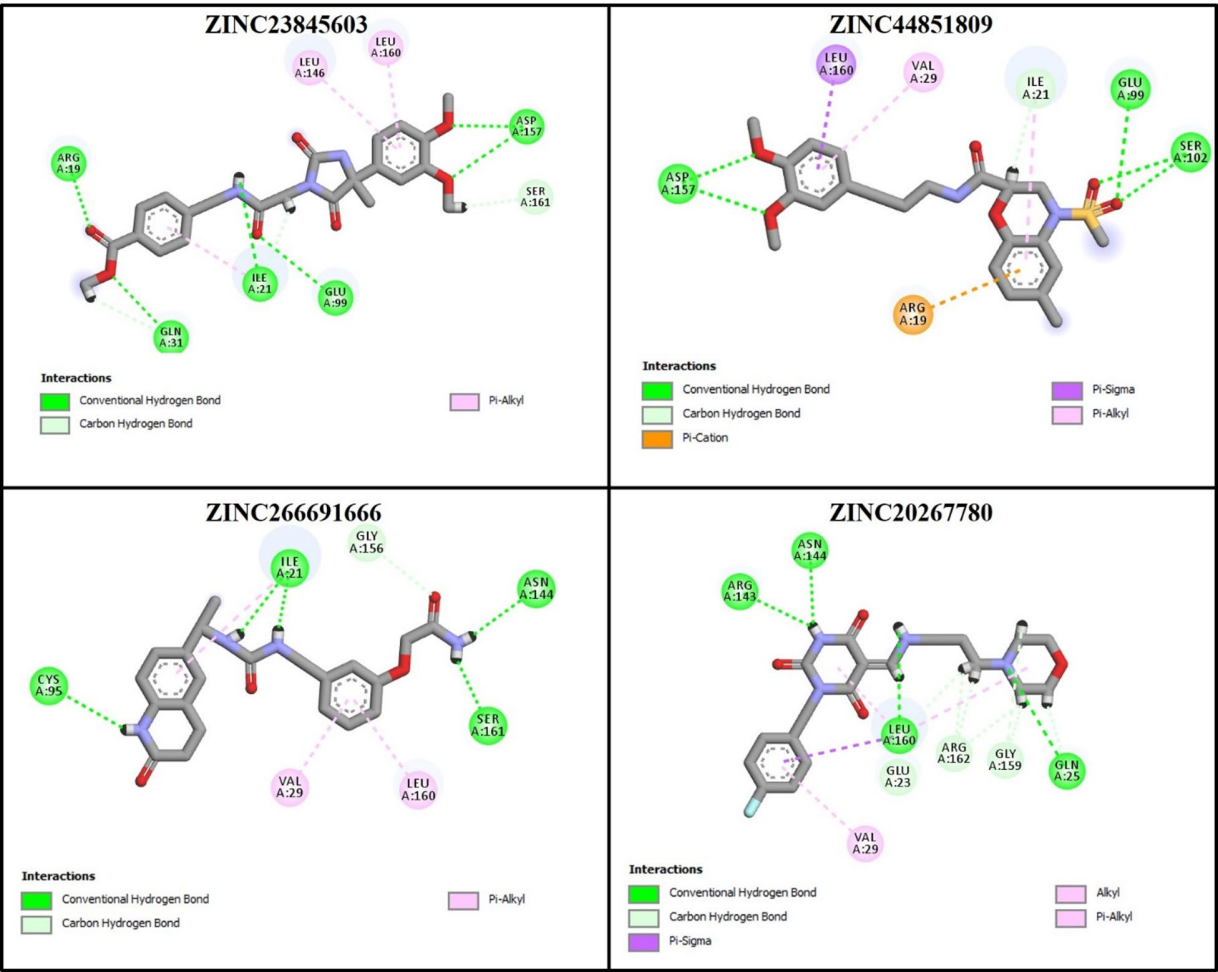
Non-bonding interactions of the selected compounds within the FAK1 binding site.

After verification of the docking method, 17 compounds with the best ADME and toxicity profiles were docked into the FAK1 kinase domain. Besides affinity, interaction diversity, particularly hydrogen bonding, were considered for the selection of the compounds in this step. The result was as follow: ZINC23845603 (−7.751 kcal/mol), ZINC44851809 (−7.932 kcal/mol), ZINC266691666 (−8.472 kcal/mol), and ZINC20267780 (−7.253 kcal/mol). Their binding modes and interaction networks are detailed in Figs. 6, 7 and 8. ZINC23845603 was stabilized by seven H-bonds (Arg19, Glu99, Asp157, Ile21, Gln31, Ser161) and π-alkyl contacts with Leu146/Leu160. ZINC44851809 engaged FAK1 via five H-bonds (Asp157, Glu99, Ser102, Ile21), π-alkyl interactions (Val29, Ile21), a π-sigma bond (Leu160), and a π-cation interaction (Arg19). ZINC266691666 formed six H-bonds (Ile21, Asn144, Ser161, Cys95, Gly156) alongside π-alkyl associations with Leu160/Val29. ZINC20267780 exhibited nine H-bonds (Arg143, Gln25, Leu160, Asn144, Arg162, Gly159, Glu23) complemented by π-alkyl (Val29, Leu160) and π-sigma (Leu160) interactions.

Further analysis shows that Ile21, Asp157, Leu160, and Arg19 are involved in all binding interactions by forming hydrogen bonds or hydrophobic interactions with the ligands.

### Molecular dynamics simulation study

MD simulation makes it possible to predict the interaction between the ligand and the receptor in a simulated aqueous environment and obtain more accurate information, such as the stability of the complex and binding energies, within this dynamic framework^46^. In this work, four compounds (ZINC23845603, ZINC44851809, ZINC266691666, ZINC20267780) and the reference ligand P4N, all bound to the FAK1 protein, were simulated under such conditions. The goal was to analyze their stability and interactions over time. The initial 20 ns simulation revealed that ZINC44851809 was unstable and dissociated from FAK1 at 16 ns. But the three other ligands, including ZINC23845603, ZINC266691666, and ZINC20267780 were stable during this time. Therefore, they were subjected to an additional 80 ns simulation (totaling 100 ns). All of them remained stable during this time as well and did not show any instability. Therefore, their simulation trajectory was further analyzed as follows.

**Fig. 9:**
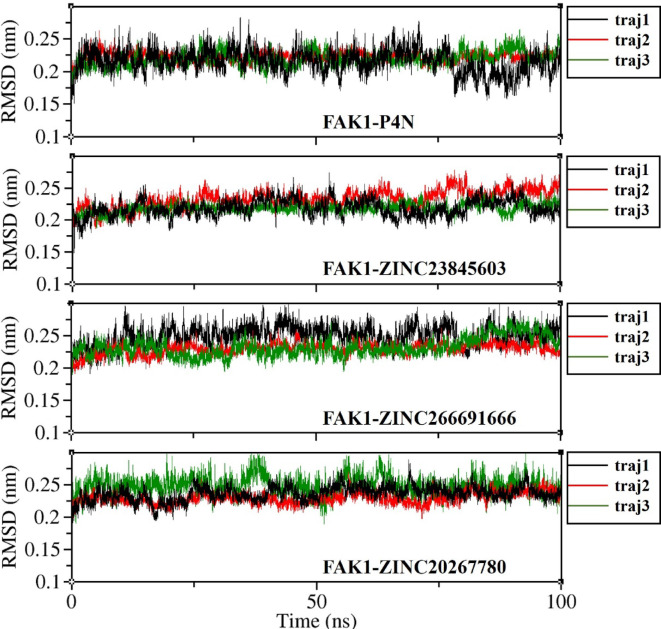
Root mean square deviation (RMSD) of the Cα atoms of FAK1 in complexes with P4N, ZINC23845603, ZINC266691666, and ZINC20267780.

**Fig. 10:**
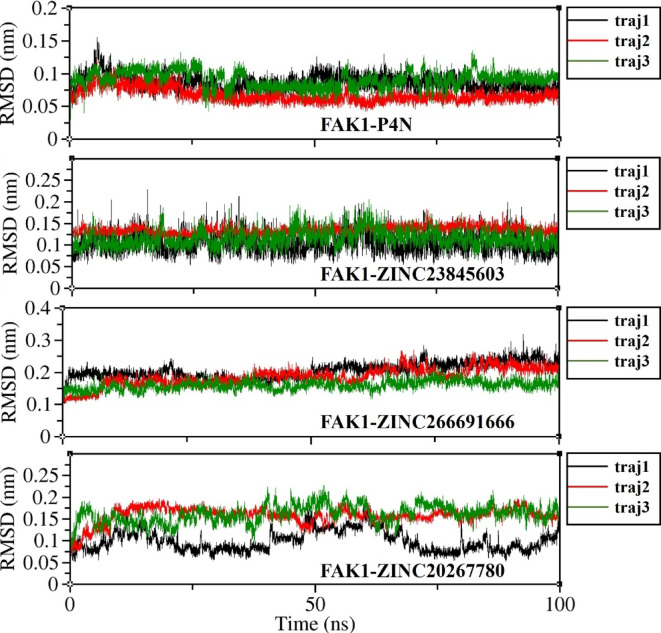
RMSD of heavy atoms of P4N, ZINC23845603, ZINC266691666, and ZINC20267780 in their complexes with FAK1.

RMSD calculation of FAK1 backbone atoms showed that it was below 3 Å throughout all 100 ns simulation time in all complexes (Fig. 9). The average RMSD of FAK1 backbone atoms was 2.18 Å in the complex with P4N and 2.22 Å, 2.36 Å and 2.37 Å in the complex with ZINC23845603, ZINC266691666, and ZINC20267780, respectively. The RMSD of all ligand atoms were under 3 Å too. The average RMSD of P4N was 0.81 Å and the average RMSD of ZINC23845603, ZINC266691666, and ZINC20267780 were 1.18 Å, 1.83 Å, and 1.39 Å, respectively (Fig. 10). This data is in favor of the stable complexes of the ligands and the FAK1 kinase domain. However, other analyses that will be discussed below also emphasize this stability.

**Fig. 11:**
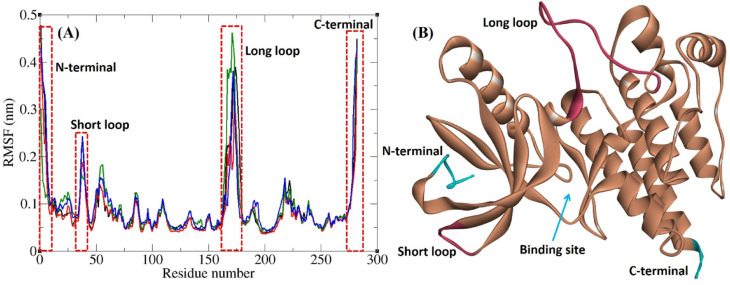
(**A**) RMSF graph of the Cα atoms of FAK1 in complexes with P4N (black), ZINC23845603 (red), ZINC266691666 (green), and ZINC20267780 (blue). (**B**) Structure of FAK1.

Flexibility of different parts of a protein can be determined by analyzing the RMSF plot. In this study, the RMSF graph of FAK1 kinase domain in complex with P4N and other ligands overlapped (Fig. 11). Besides the N-terminal and C-terminal, there were two other peaks in the RMSF plots related to loops of different sizes. The first one was related to a short loop including residues 35–45, and the next one was associated with a long loop including residues 162–180. Both of these loops are located at the surface of the FAK1 kinase domain and are exposed to water with little conformational restriction. None of the residues in these two loops were involved in ligand binding interactions. As expected, internal regions of the protein, including alpha helices and beta sheets, and residues involved in ligand binding interactions had lower RMSF values.

**Fig. 12:**
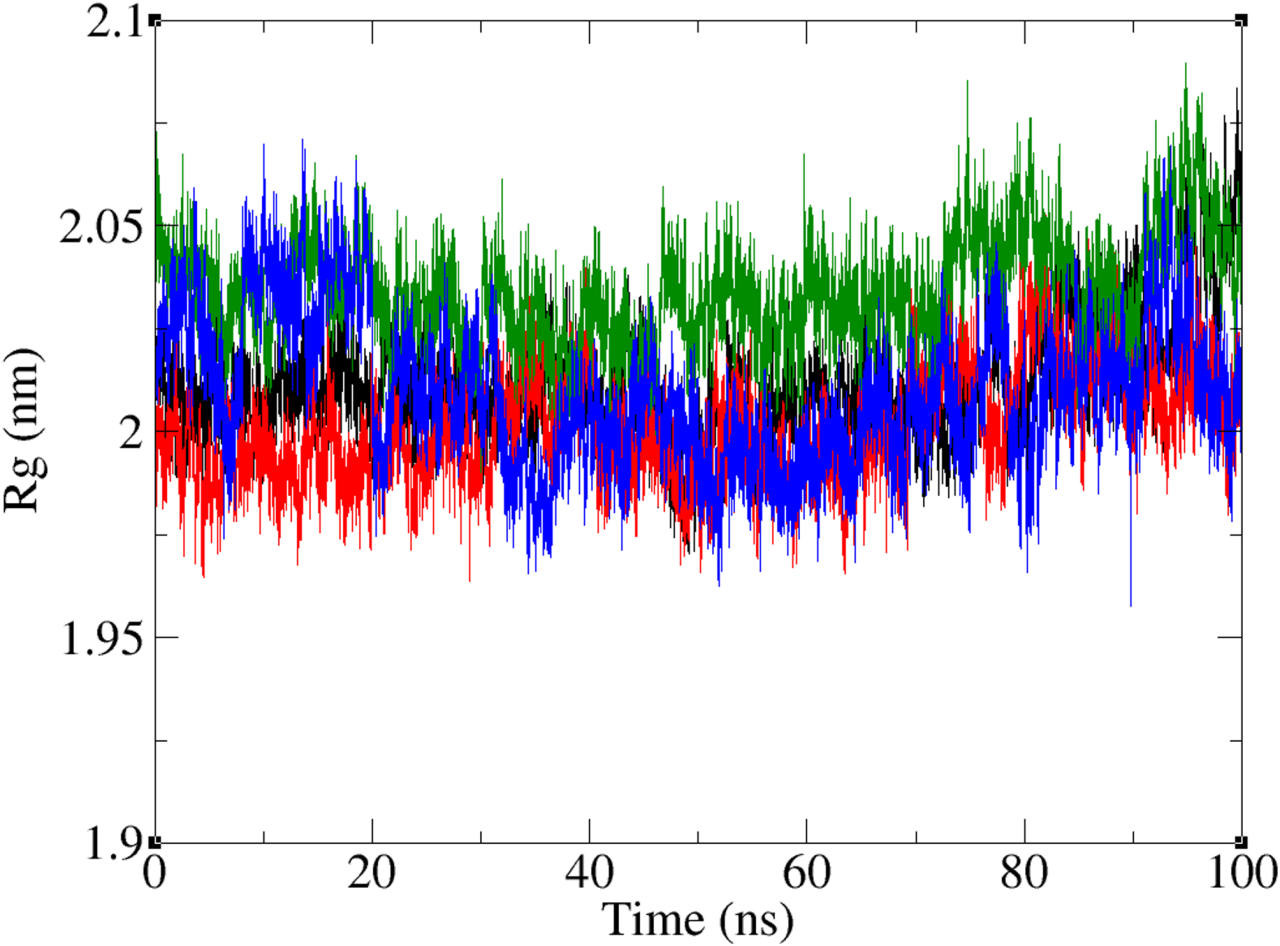
Time dependence of the radius of gyration (Rg) for FAK1 in complexes with P4N (black), ZINC23845603 (red), ZINC266691666 (green), and ZINC20267780 (blue).

Overall compactness and stability of a protein during MD simulation can be assessed by radius of gyration (Rg). Therefore, the Rg value of the FAK1 kinase domain during MD simulation was calculated for all complexes (Fig. 12). Throughout the simulations, the Rg values of all FAK1 complexes remained within a narrow range of 19.60 to 20.87 Å, which can be an indicator of stable structures. The average Rg values for FAK1 were 20.13 Å, 20.05 Å, 20.26 Å and 20.03 Å in complexes of this protein with P4N, ZINC23845603, ZINC266691666, and ZINC20267780, respectively. These results indicate that no conformational change destabilizing the FAK1 structure occurred in the presence of the lead compounds.

**Fig. 13:**
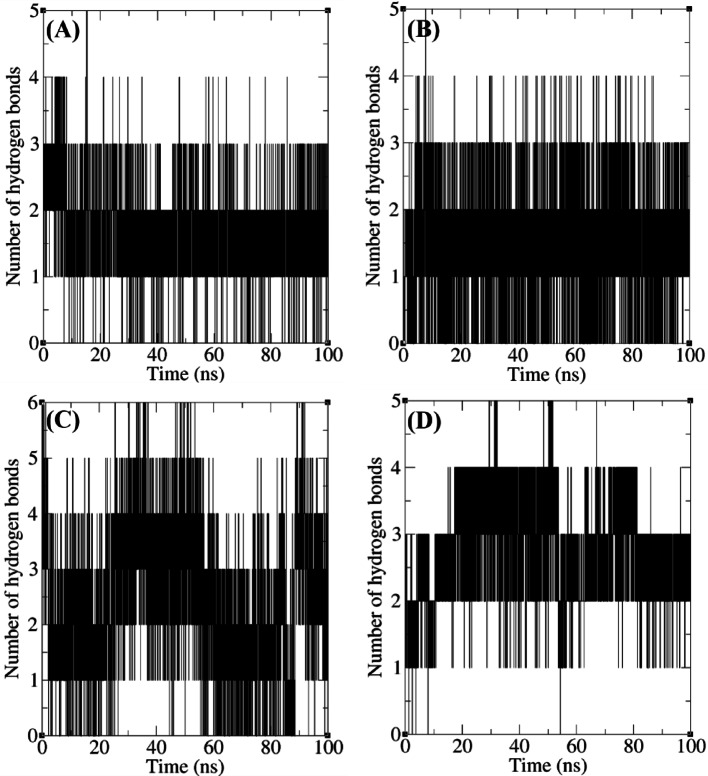
Number of hydrogen bonds formed between FAK1 and P4N (**A**), ZINC23845603 (**B**), ZINC266691666 (**C**) and ZINC20267780 (**D**).

The number of hydrogen bonds is a critical determinant of binding stability. In this study, the number of hydrogen bonds was monitored over a 100 ns trajectory (Fig. 13). The co-crystallized ligand P4N had 1–3 hydrogen bonds consistently throughout the simulation, which indicates a strong and stable interaction with FAK1. The lead compounds had various numbers of hydrogen bonds. ZINC23845603 had 1–3 hydrogen bonds, ZINC20267780 maintained 1–5 hydrogen bonds, and ZINC266691666 had 1–4 hydrogen bonds over the simulation period. However, there was no direct relationship between the number of hydrogen bonds and binding energy, which will be discussed in the following section.

### Free binding energy calculations

**Fig. 14:**
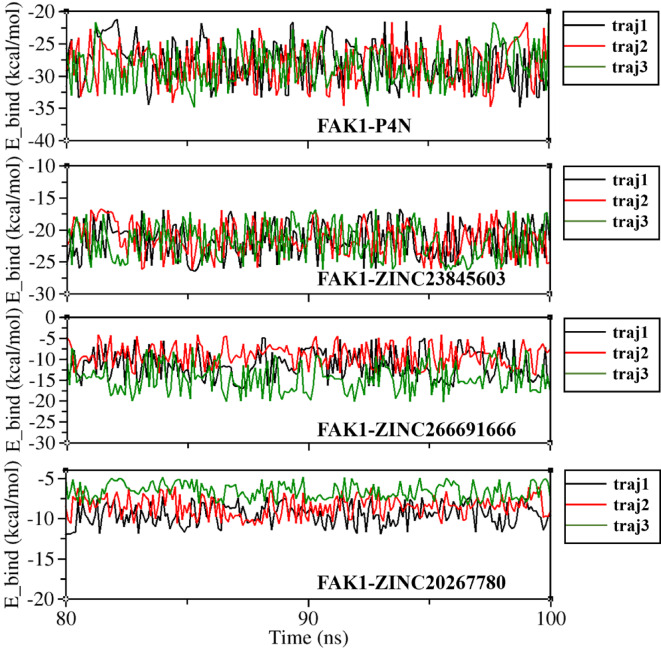
Diagram of binding energy (E_bind) fluctuations during the last 20 ns of simulation for FAK1 in complexes with P4N, ZINC23845603, ZINC266691666, and ZINC20267780.

The g_mmpbsa analysis was performed on the last 20 ns of the simulation trajectory and provided insightful data regarding the binding energy of the crystal structure ligand (P4N) and the three lead compounds (Fig. 14; Table 4). According to this analysis, all complexes were stable over the last 20 ns of the simulation. The highest binding energy belonged to P4N (−28.267 kcal/mol), which is the native ligand, evolved for optimal binding to FAK1. None of the three lead compounds dissociated during the 100 ns of MD simulation; however, ZINC20267780 and ZINC26669166 showed too weak binding energies (−8.554 and − 11.704 kcal/mol respectively), suggesting that they are less favorable candidates for further development. On the other hand, ZINC23845603 exhibited a high binding energy of −21.870 kcal/mol, fluctuating moderately around − 21 kcal/mol, which makes it a potential candidate for further optimization. Both ZINC20267780 and ZINC26669166 had higher electrostatic energy than ZINC23845603 (−8.331 and − 11.199 Kcal/mol compared to −7.051 kcal/mol), however their lower van der Waals energy (−17.774 and − 34.107 compared to −46.775 kcal/mol) led to the low total molecular mechanics interaction energy (MMIE) (−26.105 and − 45.306 kcal/mol compared to −53.826 kcal/mol). Solvation energy, which is the sum of polar solvation energy and SASA energy, was close to each other in all lead compounds (17.551 and 34.602 kcal/mol compared to 31.956 kcal/mol). Van der Waals energy was the prominent energy in all FAK1-ligand interactions, which can be because of the hydrophobic binding pocket of FAK1 kinase domain. In the P4N complex, it had the highest value (−50.858 kcal/mol), and in ZINC23845603, it was higher than ZINC20267780 and ZINC26669166 (−46.775 kcal/mol compared to −17.774 and − 34.107 respectively). The conformational flexibility and stability of the FAK1–P4N and FAK1–ZINC23845603 complexes can be further visualized in Supplementary Videos 1 and 2, described in the Supplementary Videos Legend.

**Table 4:** Binding free energy (kcal/mol) of the selected compounds and P4N.

Complex	van der Waals energy	Electrostatic energy	Polar solvation energy	SASA ^a^ energy	Binding energy
ZINC23845603	−46.775	−7.051	36.892	−4.936	−21.870
ZINC266691666	−34.107	−11.199	37.959	−4.357	−11.704
ZINC20267780	−17.774	−8.331	19.661	−2.110	−8.554
P4N	−50.858	−11.546	39.107	−4.972	−28.267

^a^ Solvent- Accessible Surface Area.

There was no direct relationship between the number of hydrogen bonds and binding energy. Binding energy was calculated during the last 20 ns of the simulation. In all complexes, the number of hydrogen bonds varied mostly between a narrow range of 1–4 during this period (Fig. 13). However, there was a significant difference between the binding energies of the complexes during this time. While P4N and ZINC23845603 had the highest binding energies (−28.267 and − 21.870 kcal/mol, respectively), the binding energies of ZINC266691666 and ZINC20267780 were only − 11.704 and − 8.554 kcal/mol, respectively. This shows that, although hydrogen bonds are important in the stability of protein-ligand complexes, forces like hydrophobic interactions, van der Waals forces, and entropic contributions also have significant roles. In g_mmpbsa, the contribution of hydrogen bonds is primarily reflected in the electrostatic energy term, with a minor contribution from van der Waals interactions. By considering more details, it was determined that despite differences between the electrostatic energies of the complexes, this difference was small (from − 7.051 to −11.546 kcal/mol), which is in accordance with the small number of hydrogen bonds in the complexes. On the other hand, van der Waals energy was the main positive contributor to the binding energy (changing from − 17.774 to −50.858 kcal/mol). Accordingly, various analyses, such as hydrogen bond monitoring and binding energy calculations, are important for obtaining a comprehensive understanding of ligand-receptor stability.

**Fig. 15:**
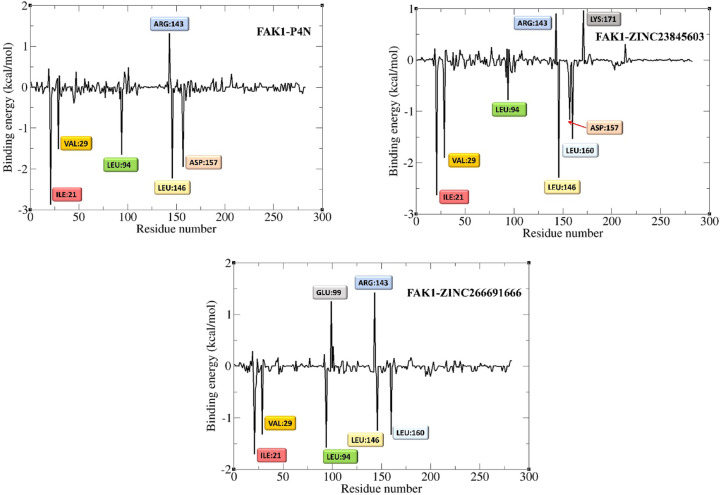
Contribution of FAK1 residues to the binding energy (kcal/mol).

**Fig. 16:**
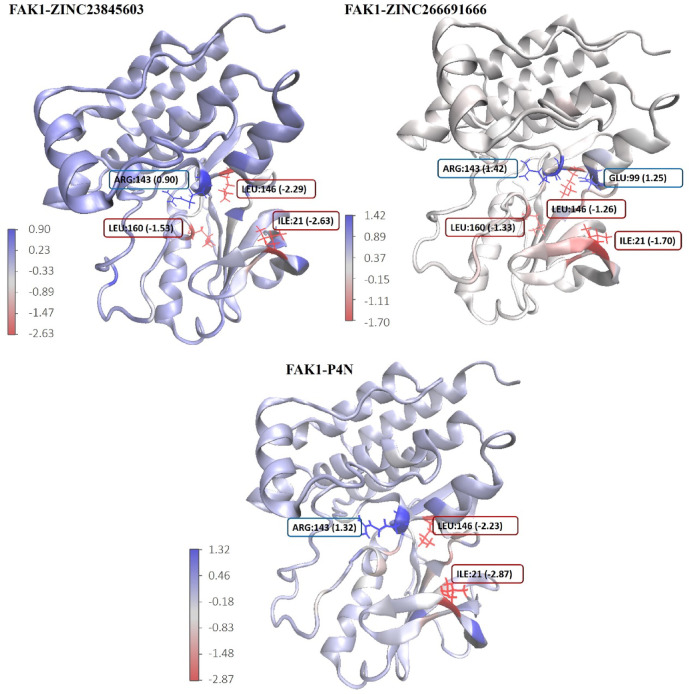
Residues with the highest and lowest contributions to the binding energy (kcal/mol).

Residue-based decomposition analysis by g_mmpbsa determines the contribution of each residue to the binding energy (Figs. 15 and 16). According to this analysis, five residues, including Leu94, Asp157, Leu146, Val29, and Ile21, had the most positive contribution to the binding energy of the P4N-FAK1 complex (Fig. 15). All these residues were involved in the binding of ZINC23845603 to FAK1 as well. They were also detected as the main residues in the docking studies (Figs. 2 and 8). The two other ligands, i.e., ZINC266691666 and ZINC20267780, did not have any interaction with most of these key residues, and this may be the reason for their low binding energies (data not shown). Besides these residues, a new residue, Lys171, was found to be important in the ZINC23845603-FAK1 complex. Moreover, Cys95, Met92, Gly156, Glu99, and Gln31, which were detected by docking studies to be important in both P4N-FAK1 and ZINC23845603-FAK1 complexes, did not have a significant contribution to the binding energy in the MD simulation studies. It is conceivable that this discrepancy is due to the dynamic nature of ligand-protein interaction and the flexibility of the protein structure. Rigid docking uses only one conformation of the thousands of conformations that a protein can possess. However, MD simulation uses several conformations during the time and calculates the binding energy and residue contribution in all these conformations in post-simulation analysis, which leads to much more accurate results.

In this study, entropy contributions were not included in the MM/PBSA calculations. MM/PBSA has well-recognized limitations due to its endpoint nature and use of an implicit solvent model^47^. However, it is widely applied for comparative binding affinity estimation. Here, the use of multiple replicas and complementary trajectory analyses strengthens the reliability of our results, despite these methodological constraints.

## Conclusion

FAK1 has become a significant target in cancer treatment as its over-activation is associated with tumor progression and metastasis. In this study, using a solid plan combining structure-based pharmacophore generation, docking, MD simulation, and MM/PBSA analyses, a new potential inhibitor of FAK1 with proper physiochemical properties and low toxicity was discovered among the ZINC chemical database. The 100 ns MD simulation trajectory of this compound, i.e., ZINC23845603, and the crystal ligand, i.e., P4N, in complex with the FAK1 kinase domain were thoroughly analyzed and compared. After calculation of binding energy parameters and analyzing the interactions of the ligands with the binding pocket residues, some important similarities were noticed. Besides having a strong binding energy, the same residues were discovered to contribute to the binding energy in both complexes, and van der Waals energy was the predominant force in the stability of the complexes. Despite being an in silico study, the findings, especially the ligand-receptor interactions, strongly support the identification of a novel and potent FAK1 inhibitor. Binding energy analyses indicate that ZINC23845603 can bind noncovalently to the active site of FAK1. This binding mode positions ZINC23845603 in direct competition with the natural substrate, thereby suggesting a competitive inhibition mechanism. Taking into account that the toxicity and pharmacokinetic predictions were also in favor of this compound, in follow-up studies, both in vitro and in vivo assessments will be performed on ZINC23845603 to verify the predictions.

## Supplementary Information

Below is the link to the electronic supplementary material.

Supplementary Material 1

Supplementary Material 2

Supplementary Material 3

## Data Availability

The datasets generated during and/or analysed during the current study are available from the corresponding author on reasonable request.

## References

[1] Le Coq, J. et al. *New insights into FAK structure and function in focal adhesions*. *J. Cell. Sci.*, **135**(20), jcs259089 (2022). 10.1242/jcs.259089 36239192

[2] Levy, A. et al. Focal adhesion kinase in ovarian cancer: A potential therapeutic target for platinum and Taxane-Resistant tumors. *Curr. Cancer Drug Targets*. **19** (3), 179–188 (2019). 29984656 10.2174/1568009618666180706165222

[3] Sulzmaier, F. J., Jean, C. & Schlaepfer, D. D. FAK in cancer: mechanistic findings and clinical applications. *Nat. Rev. Cancer*. **14** (9), 598–610 (2014). 25098269 10.1038/nrc3792 PMC4365862

[4] Aboubakar Nana, F., Vanderputten, M. & Ocak, S. *Role of focal adhesion kinase in Small-Cell lung cancer and its potential as a therapeutic target*. *Cancers (Basel)*, **11**(11), 1683 (2019). 10.3390/cancers11111683 PMC6895835 31671774

[5] Lietha, D. et al. Structural basis for the autoinhibition of focal adhesion kinase. *Cell* **129** (6), 1177–1187 (2007). 17574028 10.1016/j.cell.2007.05.041 PMC2077847

[6] Jones, S. F. et al. A phase I study of VS-6063, a second-generation focal adhesion kinase inhibitor, in patients with advanced solid tumors. *Invest. New. Drugs*. **33** (5), 1100–1107 (2015). 26334219 10.1007/s10637-015-0282-y

[7] Golubovskaya, V. M. et al. A small molecule focal adhesion kinase (FAK) inhibitor, targeting Y397 site: 1-(2-hydroxyethyl)-3, 5, 7-triaza-1-azoniatricyclo [3.3.1.1(3,7)]decane; bromide effectively inhibits FAK autophosphorylation activity and decreases cancer cell viability, clonogenicity and tumor growth in vivo. *Carcinogenesis* **33** (5), p1004–p1013 (2012). 10.1093/carcin/bgs120 PMC3334519 22402131

[8] Lv, P., Chen, K. & Zhu, H. L. Recent advances of small molecule focal adhesion kinase (FAK) inhibitors as promising anticancer therapeutics. *Curr. Med. Chem.* **28** (34), 6977–6989 (2021). 33797359 10.2174/0929867328666210331143827

[9] Wu, Y. et al. Focal adhesion kinase inhibitors, a heavy punch to cancer. *Discov Oncol.* **12** (1), 52 (2021). 35201485 10.1007/s12672-021-00449-y PMC8777493

[10] Brullo, C. & Tasso, B. New insights on Fak and Fak inhibitors. *Curr. Med. Chem.* **28** (17), 3318–3338 (2021). 33143618 10.2174/0929867327666201103162239

[11] Koide, E. et al. Development and characterization of selective FAK inhibitors and protacs with in vivo activity. *Chembiochem* **24** (19), e202300141 (2023). 37088717 10.1002/cbic.202300141 PMC10590827

[12] Khan, A. et al. Repositioning of experimentally validated anti-breast cancer peptides to target FAK-PAX complex to halt the breast cancer progression: a biomolecular simulation approach. *Mol. Divers.* **27** (2), 603–618 (2023). 35635599 10.1007/s11030-022-10438-0

[13] Furqan, M. et al. *Drug Combinations Targeting FAK and MEK Overcomes Tumor Heterogeneity in Glioblastoma.* 10.3390/pharmaceutics17050549 PMC12114623 40430842

[14] Banerjee, S. N. et al. Initial efficacy and safety results from ENGOT-ov60/GOG-3052/RAMP 201: A phase 2 study of Avutometinib (VS-6766) ± defactinib in recurrent low-grade serous ovarian cancer (LGSOC). *J. Clin. Oncol.* **41** (16_suppl), 5515–5515 (2023).

[15] Cha, J. & Kim, P. Cancer cell-Sticky hydrogels to target the cell membrane of invading glioblastomas. *ACS Appl. Mater. Interfaces*. **13** (27), 31371–31378 (2021). 34196172 10.1021/acsami.1c00388

[16] Kumar, V. et al. Computational insights into allosteric Inhibition of focal adhesion kinase: A combined pharmacophore modeling and molecular dynamics approach. *J. Mol. Graph Model.* **130**, 108789 (2024). 38718434 10.1016/j.jmgm.2024.108789

[17] Shi, M. et al. Molecular Docking, molecular dynamics Simulations, and free energy calculation insights into the binding mechanism between VS-4718 and focal adhesion kinase. *ACS Omega*. **7** (36), 32442–32456 (2022). 36119979 10.1021/acsomega.2c03951 PMC9476166

[18] Lu, Y. & Sun, H. Progress in the development of small molecular inhibitors of focal adhesion kinase (FAK). *J. Med. Chem.* **63** (23), 14382–14403 (2020). 33058670 10.1021/acs.jmedchem.0c01248

[19] Paradis, J. S. et al. Synthetic lethal screens reveal cotargeting FAK and MEK as a multimodal precision therapy for GNAQ-Driven uveal melanoma. *Clin. Cancer Res.* **27** (11), 3190–3200 (2021). 33568347 10.1158/1078-0432.CCR-20-3363 PMC8895627

[20] Liu, H. et al. Doxycycline inhibits cancer stem Cell-Like properties via PAR1/FAK/PI3K/AKT pathway in pancreatic cancer. *Front. Oncol.* **10**, 619317 (2020). 33643917 10.3389/fonc.2020.619317 PMC7905084

[21] Aakriti, J. et al. Focal adhesion kinase (FAK): emerging target for drug-resistant malignant tumors. *Mol. Biol. Rep.* **52** (1), 248 (2025). 39976799 10.1007/s11033-025-10296-7 PMC11842479

[22] Gimeno, A. et al. *The light and dark sides of virtual screening: what is there to know?*. *Int. J. Mol. Sci.*, **20**(6), 1375 (2019). 10.3390/ijms20061375 PMC6470506 30893780

[23] Kar, S. & Roy, K. How Far can virtual screening take Us in drug discovery? *Expert Opin. Drug Discov*. **8** (3), 245–261 (2013). 23330660 10.1517/17460441.2013.761204

[24] Lin, X., Li, X. & Lin, X. *A Rev. Appl. Comput. Methods Drug Screen. Des. Molecules*, **25**(6), 1375 (2020).

[25] Singh, H. & Bharadvaja, N. Treasuring the computational approach in medicinal plant research. *Prog Biophys. Mol. Biol.* **164**, 19–32 (2021). 34004233 10.1016/j.pbiomolbio.2021.05.004

[26] Naqvi, A. A. T. et al. Advancements in Docking and molecular dynamics simulations towards Ligand-receptor interactions and Structure-function relationships. *Curr. Top. Med. Chem.* **18** (20), 1755–1768 (2018). 30360721 10.2174/1568026618666181025114157

[27] Sledz, P. & Caflisch, A. Protein structure-based drug design: from Docking to molecular dynamics. *Curr. Opin. Struct. Biol.* **48**, 93–102 (2018). 29149726 10.1016/j.sbi.2017.10.010

[28] Berger, B. T. et al. Structure-kinetic relationship reveals the mechanism of selectivity of FAK inhibitors over PYK2. *Cell. Chem. Biol.* **28** (5), 686–698e7 (2021). 33497606 10.1016/j.chembiol.2021.01.003

[29] Webb, B. & Sali, A. Comparative protein structure modeling using MODELLER. *Curr. Protoc. Bioinf.* **54** (p. 5 6 1–5), 637 (2016). 10.1002/cpbi.3 PMC5031415 27322406

[30] Webb, B. & Sali, A. Comparative protein structure modeling using MODELLER. *Curr. Protoc. Bioinf.* **47**, 1–32 (2014). 10.1002/0471250953.bi0506s47 25199792

[31] Fiser, A., Do, R. K. & Sali, A. Modeling of loops in protein structures. *Protein Sci.* **9** (9), 1753–1773 (2000). 11045621 10.1110/ps.9.9.1753 PMC2144714

[32] Giordano, D. et al. *Drug design by pharmacophore and virtual screening approach*. *Pharmaceuticals (Basel)*, **15**(5), 646 (2022). 10.3390/ph15050646 PMC9145410 35631472

[33] Vuorinen, A. & Schuster, D. Methods for generating and applying pharmacophore models as virtual screening filters and for bioactivity profiling. *Methods* **71**, 113–134 (2015). 25461773 10.1016/j.ymeth.2014.10.013

[34] Mysinger, M. M. et al. Directory of useful decoys, enhanced (DUD-E): better ligands and decoys for better benchmarking. *J. Med. Chem.* **55** (14), 6582–6594 (2012). 22716043 10.1021/jm300687e PMC3405771

[35] Braga, R. C. & Andrade, C. H. Assessing the performance of 3D pharmacophore models in virtual screening: how good are they? *Curr. Top. Med. Chem.* **13** (9), 1127–1138 (2013). 23651486 10.2174/1568026611313090010

[36] Yang, J. M. et al. Consensus scoring criteria for improving enrichment in virtual screening. *J. Chem. Inf. Model.* **45** (4), 1134–1146 (2005). 16045308 10.1021/ci050034w

[37] Ferreira, L. L. G. & Andricopulo, A. D. ADMET modeling approaches in drug discovery. *Drug Discov Today*. **24** (5), 1157–1165 (2019). 30890362 10.1016/j.drudis.2019.03.015

[38] Daina, A., Michielin, O. & Zoete, V. SwissADME: a free web tool to evaluate pharmacokinetics, drug-likeness and medicinal chemistry friendliness of small molecules. *Sci. Rep.* **7**, 42717 (2017). 28256516 10.1038/srep42717 PMC5335600

[39] Banerjee, P. et al. ProTox-II: a webserver for the prediction of toxicity of chemicals. *Nucleic Acids Res.* **46** (W1), W257–W263 (2018). 29718510 10.1093/nar/gky318 PMC6031011

[40] Grosdidier, A., Zoete, V. & Michielin, O. *SwissDock, a protein-small molecule Docking web service based on EADock DSS*. *Nucleic Acids Res*. 39, W270–W277 (2011). 10.1093/nar/gkr366 PMC3125772 21624888

[41] Grosdidier, A., Zoete, V. & Michielin, O. Fast Docking using the CHARMM force field with EADock DSS. *J. Comput. Chem.* **32** (10), 2149–2159 (2011). 21541955 10.1002/jcc.21797

[42] Hanwell, M. D. et al. Avogadro: an advanced semantic chemical editor, visualization, and analysis platform. *J. Cheminform*. **4** (1), 17 (2012). 22889332 10.1186/1758-2946-4-17 PMC3542060

[43] Pettersen, E. F. et al. UCSF Chimera–a visualization system for exploratory research and analysis. *J. Comput. Chem.* **25** (13), 1605–1612 (2004). 15264254 10.1002/jcc.20084

[44] Abraham, M. J. et al. GROMACS: high performance molecular simulations through multi-level parallelism from laptops to supercomputers. *SoftwareX* **1-2**, 19–25 (2015).

[45] Zoete, V. et al. SwissParam: a fast force field generation tool for small organic molecules. *J. Comput. Chem.* **32** (11), 2359–2368 (2011). 21541964 10.1002/jcc.21816

[46] Karplus, M. & McCammon, J. A. Molecular dynamics simulations of biomolecules. *Nat. Struct. Biol.* **9** (9), 646–652 (2002). 12198485 10.1038/nsb0902-646

[47] Roux, B. & Chipot, C. Editorial guidelines for computational studies of ligand binding using MM/PBSA and MM/GBSA approximations wisely. *J. Phys. Chem. B*. **128** (49), 12027–12029 (2024). 39620637 10.1021/acs.jpcb.4c06614

