# Supplementary material for: Structure-based identification of novel FAK1 inhibitors using pharmacophore modeling, molecular dynamics, and MM/PBSA calculations
Source: Sci Rep. 2025 Nov 11;15:39506. doi: 10.1038/s41598-025-23203-8 (PMC12606203; doi:10.1038/s41598-025-23203-8)
Supplement: Supplementary file 1 — Supplementary Material 1 [file 41598_2025_23203_MOESM1_ESM.docx]

**Supplementary Videos Legend:**

**Video 1: FAK1-P4N_MD Simulation (100 ns)**

This video shows the 100 ns molecular dynamics simulation of FAK1 in complex with the crystal ligand P4N. The FAK1 protein is shown in green, while the carbon atoms of the P4N molecule are in orange. The video illustrates the conformational dynamics of the FAK1-P4N complex, providing insight into the stability and flexibility of the protein-ligand system over time.

**Video 2: FAK1-ZINC23845603_MD Simulation (100 ns)**

This video demonstrates the 100 ns molecular dynamics simulation of FAK1 bound to ZINC23845603. FAK1 is represented in green, and the inhibitor's carbon atoms are depicted in orange. The video showcases the dynamic behavior of the FAK1-ZINC23845603 complex, emphasizing the overall stability and conformational changes of the system throughout the simulation.
